# Dexamethasone rescues TGF-β1-mediated β_2_-adrenergic receptor dysfunction and attenuates phosphodiesterase 4D expression in human airway smooth muscle cells

**DOI:** 10.1186/s12931-020-01522-w

**Published:** 2020-10-08

**Authors:** Elena Chung, Christie A. Ojiaku, Gaoyuan Cao, Vishal Parikh, Brian Deeney, Shengjie Xu, Serena Wang, Reynold A. Panettieri, Cynthia Koziol-White

**Affiliations:** 1 Department of Pharmacology and Toxicology, School of Pharmacy, EOHSI, Rutgers University, Piscataway, NJ USA; 2 Rutgers Institute for Translational Medicine and Science, Rutgers, The State University of New Jersey, New Brunswick, NJ USA

**Keywords:** Glucocorticoids, Severe asthma, Bronchodilation, Airway remodeling

## Abstract

Glucocorticoids (GCs) and β_2_-adrenergic receptor (β_2_AR) agonists improve asthma outcomes in most patients. GCs also modulate gene expression in human airway smooth muscle (HASM), thereby attenuating airway inflammation and airway hyperresponsiveness that define asthma. Our previous studies showed that the pro-fibrotic cytokine, transforming growth factor- β1 (TGF-β1) increases phosphodiesterase 4D (PDE4D) expression that attenuates agonist-induced levels of intracellular cAMP. Decreased cAMP levels then diminishes β_2_ agonist-induced airway relaxation. In the current study, we investigated whether glucocorticoids reverse TGF-β1-effects on β_2_-agonist-induced bronchodilation and modulate *pde4d* gene expression in HASM. Dexamethasone (DEX) reversed TGF-β1 effects on cAMP levels induced by isoproterenol (ISO). TGF-β1 also attenuated G protein-dependent responses to cholera toxin (CTX), a G_αs_ stimulator downstream from the β_2_AR receptor. Previously, we demonstrated that TGF-β1 treatment increased β_2_AR phosphorylation to induce hyporesponsiveness to a β_2_ agonist. Our current data shows that expression of *grk2/3*, kinases associated with attenuation of β_2_AR function, are not altered with TGF-β1 stimulation. Interestingly, DEX also attenuated TGF-β1-induced *pde4d* gene expression. These data suggest that steroids may be an effective therapy for treatment of asthma patients whose disease is primarily driven by elevated TGF-β1 levels.

## Clinical relevance

TGF-β1 has been identifed as pivotal in mediating airway remodeling and irreversible airway obstruction. Inhaled GCs and β_2_AR agonists are commonly used to treat asthma. Our study, however, demonstrates a protective effect of dexamethasone on TGF-β1-mediated attenuation of β_2_-agonist and G_αs_-dependent relaxation of airway smooth muscle.

## Background

Asthma, a chronic inflammatory disease of the lungs manifests by several hallmarks: airway hyperresponsiveness, remodeling, and inflammation [1]. Airway smooth muscle (ASM) cells play an integral role in regulating bronchomotor tone in the asthma diatheses and are a direct target of β_2_-agonists, a common therapy that promotes bronchodilation [2]. While β_2_-agonists evoke ASM relaxation, β2-agonists are not effective in all patients [3]. Patients who fall into the “severe” category of asthma are frequently hyporesponsive to bronchodilators [4]. Studies show that β_2_AR tolerance or desensitization occurs after repeated bronchodilator use that diminishes drug efficacy [3, 5–9].

TGF-β1, a profibrotic cytokine whose levels are elevated in patients with asthma, augments human airway smooth muscle (HASM) cell stiffness and significantly increases myosin light chain (MLC) phosphorylation via Smad3 [10] that enhance contractile agonist-induced cell shortening and hyperresponsiveness. In addition to amplifying bronchoconstriction, we also demonstrated that TGF-β1 blunts intracellular cAMP by upregulating *pde4d* expression that decreases β_2_-agonist-induced cAMP levels [11].

Signaling downstream of seven transmembrane G protein-coupled receptors (GPCRs) involves Gαβγ trimer dissociation following receptor activation [12]. The G_α_ subunit family is comprised of G_αi_, G_αq_, and G_αs_, playing fundamental roles in regulating HASM relaxation and contraction [13]. Cholera toxin (CTX) catalyzes the ADP-ribosylation of G_αs_ that elicits adenylyl cyclase (AC) activation, causing the accumulation of intracellular cAMP and further activation of PKA to induce HASM relaxation [14]. CTX, and the β_2−_agonist isoproterenol (ISO), induce actin depolymerization in HASM in PKA-independent and -dependent pathways integrating activation of Src protein tyrosine kinases and G_αs_ protein [15], to promote smooth muscle relaxation. Whether TGF-β1 directly modulates G_αs_ protein activation remains unknown.

Glucocorticoids (GCs) remain a cornerstone in the management of asthma. GC treatment alters gene expression in HASM, thereby modulating inflammation and airway reactivity. Others have demonstrated that dexamethasone, a glucocorticoid, can directly inhibit Smad3 activity [16, 17]. Our previous study showed that TGF-β1 blunted the effects of β_2_ agonist-induced reversal of carbachol-mediated phosphorylation of myosin light chain, a process which was Smad3-dependent [11]. Given this information, we posited that GC treatment would reverse TGF-β1-induced hyporesponsiveness to β_2_-agonist. Our data demonstrate that dexamethasone (DEX) reverses TGF-β1-induced attenuation of β_2_AR-induced signaling, rescuing β_2_-agonist- and G_αs_-activator-mediated cAMP production by attenuating *pde4d* expression.

## Methods

### HASM cell culture

HASM cells from the National Disease Research Interchange (Philadelphia, PA, USA) and the International Institute for the Advancement of Medicine (Edison, NJ, USA) were derived from trachea obtained from donors without chronic illness. All tissue was obtained from de-identified donors and was deemed non-human subjects research by the Rutgers University Institutional Review Board. Cells were cultured in Ham’s F12 medium with 10% fetal bovine serum. The cells were incubated and grown at 37 °C in 5% CO_2_. We have shown that isolated airway smooth muscle cells retain their phenotypic properties [18]. Primary HASM cells between passages 3–4 were used in all experiments. Donor demographics for the cell lines utilized in these studies are detailed in Table S1.

### Western blot analysis

Primary HASM cells were serum deprived for 24 h prior to treatment. HASM cells were lysed with 0.6 N HClO_4_, scraped, collected, and pelleted as previously described [19]. The membrane was blocked with ready-to-use Odyssey Blocking Buffer (LI-COR BioSciences) containing 0.1% sodium azide and probed for phospho-Smad3, pMLC, total MLC, and GAPDH.

### Measurement of intracellular cAMP levels

Grown to 90% confluency on 24-well plates, HASM cells were stimulated and lysed using the Applied Biosystems cAMP-Screen Immunoassay System following the manufacturer instructions as previously described [11]. The cells were lysed and incubated for 30 min in 5% CO_2_ and 37 °C. Conjugate Dilution Buffer, cAMP-AP Conjugate, anti-cAMP antibody, and the samples were added to pre-coated assay plate to incubate for 1 h on plate shaker. Plate was measured on luminometer after a 30-min incubation period with CSPD®/Sapphire-II RTU Substrate. Data was derived from standard curves and cAMP levels reported after using standard dilutions.

### Quantification of *pde4d, grk2, and grk3* expression (RNA isolation and qPCR)

Following treatment with TGF-β1 ± dexamethasone, cells were suspended in TRIzol reagent, and total RNA were isolated following the manufacturer protocol. RNA was isolated and purified from HASM cells using the RNeasy Mini Kit and cDNA was created using SuperScript IV First-Strand Synthesis System. All reactions were performed in 20 μL reaction volume in triplicate. For mRNA cDNA, PCR amplification consisted of 10 min of an initial denaturation step at 95 °C, followed by 40 cycles at 95 °C for 15 s, 60 °C for 60 s. Relative cDNA quantification was performed using TaqMan quantitative RT-PCR (Thermo Fisher Scientific) and the ΔΔC_t_ method, and *pde4d, grk2, and grk3* expression were normalized to expression of endogenous *β-actin*.

### Statistical analysis

Graphs were created and statistical analyses were conducted using GraphPad Prism 5.01 h software (La Jolla, Ca, USA) to determine statistical significance evaluated using two-tailed Student’s paired *t*-test for two groups. *P* values of < 0.05 were considered significant. All results were confirmed by experiments in at least three unique cell lines. Data was fit to a normal distribution, and appropriate tests run to determine significance. For comparison of multiple conditions, one-way ANOVA was used with Bonferroni’s post-test. For the *pde4d* expression results, the differential expression analysis was performed under a negative binomial distribution model with DESeq2 (v.1.18.1), and the adjusted *p* values are noted.

### Materials

Compounds were purchased from the following vendors: R&D Systems (TGF-β1; SB-431542), Sigma-Aldrich (albuterol [Alb], carbachol [Cch], cholera toxin [CTX], dexamethasone [DEX], isoproterenol [ISO]), Fisher BioReagents (Forskolin, [FSK]). Immunoblot antibodies were purchased from Abcam (phospho-Smad3; ab52903), Cell Signaling Technologies (phosphorylated myosin light chain pMLC, 3674S; GAPDH, 2118S; Tubulin, 3873S), and EMD Millipore (total myosin light chain [MLC, MABT180]). The following Taqman primer sets were purchased from Thermo Fisher Scientific: ACTB, actin beta, Hs01060665_g1; GRK2, G protein-coupled receptor kinase 2 Hs00176395_m1; GRK3, G protein-coupled receptor kinase 3, Hs00178266_m1; PDE4D, phosphodiesterase 4D, Hs01579625_m1.

## Results

### TGF-β1 attenuates G_αs_-mediated cAMP production in HASM

**Fig. 1:**
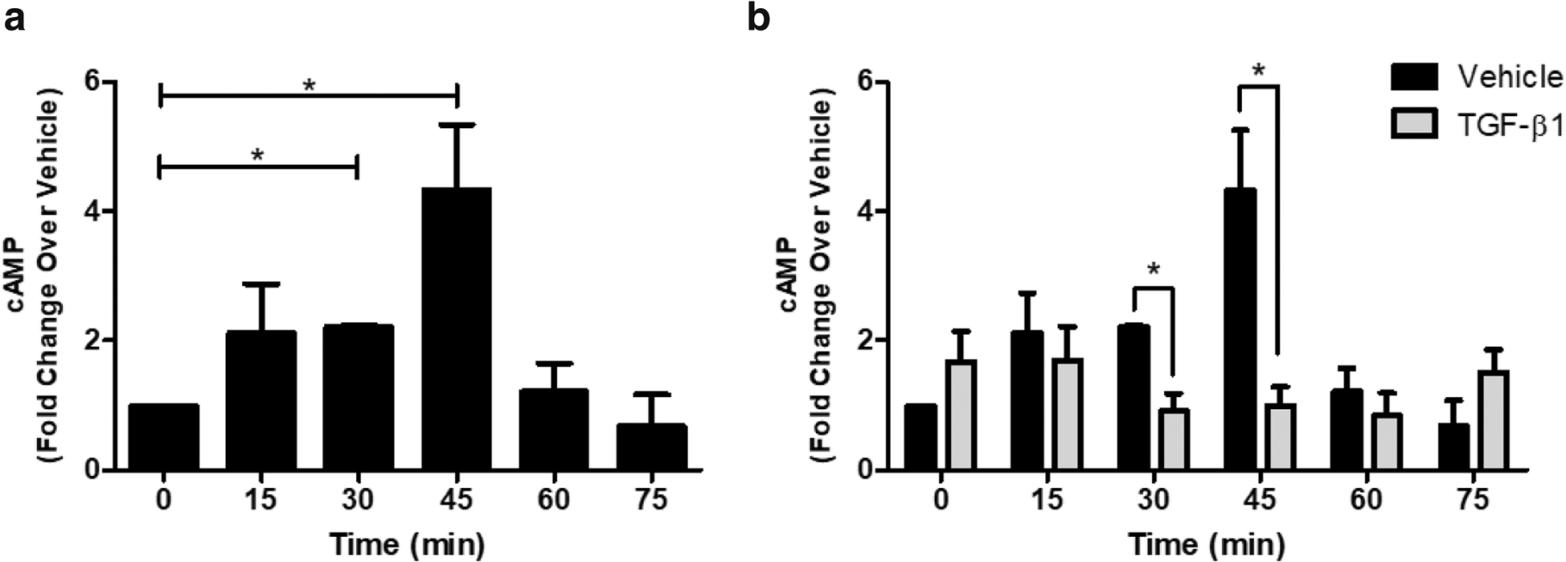
Cholera toxin (CTX) increases cAMP levels in HASM, which is blunted by overnight treatment with TGF-β1. A time course of CTX-, a G_αs_ activator, induced cAMP production in HASM (0.25 μg/ml, 0–75 min) was performed in the presence and absence of TGF-β1 (10 ng/ml, 18 h). Data is expressed fold change over vehicle control as mean ± SEM for *n* = 3 separate cell lines, three additional donors were added to the 45 min to confirm appropriate time point. *p < 0.05 as assessed by one-way ANOVA, and comparisons between two conditions assessed by Student’s t-test

Upon activation of β_2_AR, ADP-ribosylation of the α subunit of stimulatory G protein (G_αs_), stimulates adenylyl cyclase to increase intracellular cAMP [15, 19]. To further understand mechanisms underlying TGF-β1-mediated hyporesponsiveness to a β_2_-agonist, intracellular cAMP levels were measured in TGF-β1-treated HASM cells following stimulation with cholera toxin (CTX), a G_αs_ activator. Intracellular cAMP activity increased in a time-dependent manner following exposure to CTX, with the maximum level elicited at 45 min (Fig. 1a). Interestingly at 60 and 75 min, the CTX-induced cAMP levels extinguished. In TGF-β1-treated cells, CTX-induced cAMP levels were completely abrogated compared to that of the diluent control (Fig. 1b).

**Fig. 2:**
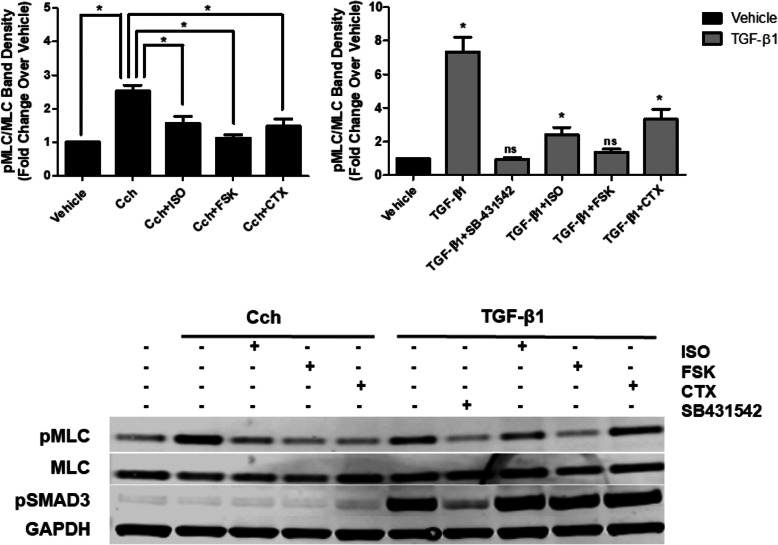
Overnight TGF-β1 treatment impairs CTX-induced MLC dephosphorylation in HASM. Phosphorylation of myosin light chain (pMLC) following Cch (20 μM, 13 min) or TGF-β1 (10 ng/ml, 18 h) or was assessed following ISO (1 μM, 10 min), FSK (10 μM, 15 min), or CTX (0.25 μg/ml, 45 min) treatment. SB-431542 (5 μM, 1 h prior to TGF-β1 treatment), a TGF-β1 receptor inhibitor, was used as a control. All treatments (ISO/FSK/SB/CTX) significantly attenuated TGF-β1-induced pMLC (*p* < 0.05). Phosphorylation of MLC was normalized to total MLC for each experiment. *p < 0.05 as assessed by one-way ANOVA, and comparisons between two conditions assessed by Student’s t-test. Data is representative of *n* = 5–6 distinct HASM cell lines

We previously demonstrated that isoproterenol (ISO) decreased carbachol (Cch)- and TGF-β1-induced phosphorylation of myosin light chain (pMLC); TGF-β1, however, decreased the ability of a β_2_-agonist to abrogate Cch-induced pMLC [11]. Cch-induced pMLC was inhibited by ISO, forskolin (an adenylyl cyclase activator) and CTX to comparable levels, as shown in Fig. 2. Interestingly, TGF-β1-induced pMLC was also decreased with CTX, forskolin, ISO and SB-431542, a TGF-β1 receptor antagonist. However, ISO and CTX-induced inhibition was less effective as compared with that of SB-431542 or forskolin in blocking TGF-β-induced pMLC (Fig. 2, upper right). Phosphorylation of SMAD3 (pSMAD3) induced by TGF-β1 confirmed engagement of TGF-β1 receptors and activation of downstream signaling pathways (Fig. 2, lower panel). Collectively, these data suggest that TGF-β1 inhibits the ability of β2AR or G_αs_ activation, but not forskolin, to increase cAMP levels and diminish pMLC.

### TGF-β1 attenuates *grk2* and *grk3* expression in HASM

**Fig. 3:**
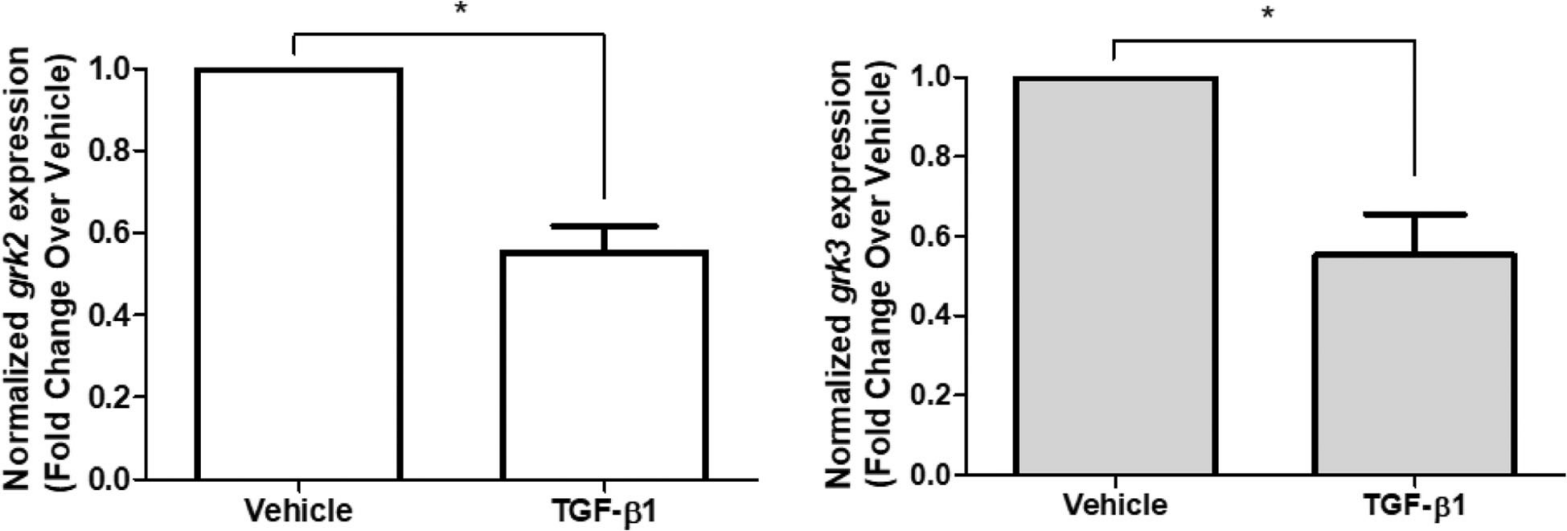
TGF-β1 attenuates expression of *grk2* and *grk3*. HASM were treated with TGF-β1 (10 ng/ml, 18 h), total RNA was isolated, and gene expression was assessed by TaqMan qPCR. Expression of *grk2* and *grk3* was normalized to endogenous *β-actin.* Data is representative of *n* = 5–6 different donors as mean ± SEM, *p < 0.05 by Student’s t-test

One mechanism by which inflammatory mediators can attenuate signaling downstream of the β_2_AR is through phosphorylation of the receptor [20–23]. Evidence suggests that GRK2 and 3 are associated with the β_2_AR, mediating desensitization through phosphorylation of intracellular portions of the receptor [24, 25]. We next examined the effect of TGF-β1 on expression of GRK2 and 3. As shown in Fig. 3, we show that TGF-β1 attenuates, rather than augments, *grk2* and *grk3* expression. Despite our previous findings showing that β_2_AR phosphorylation is increased following TGF-β1 exposure [26], we determined that the increase in TGF-β1-induced β_2_AR phosphorylation is not due to increased expression of GRK2/3.

### Dexamethasone rescues TGF-β1-induced decreases in cAMP levels induced by ISO or CTX

**Fig. 4:**
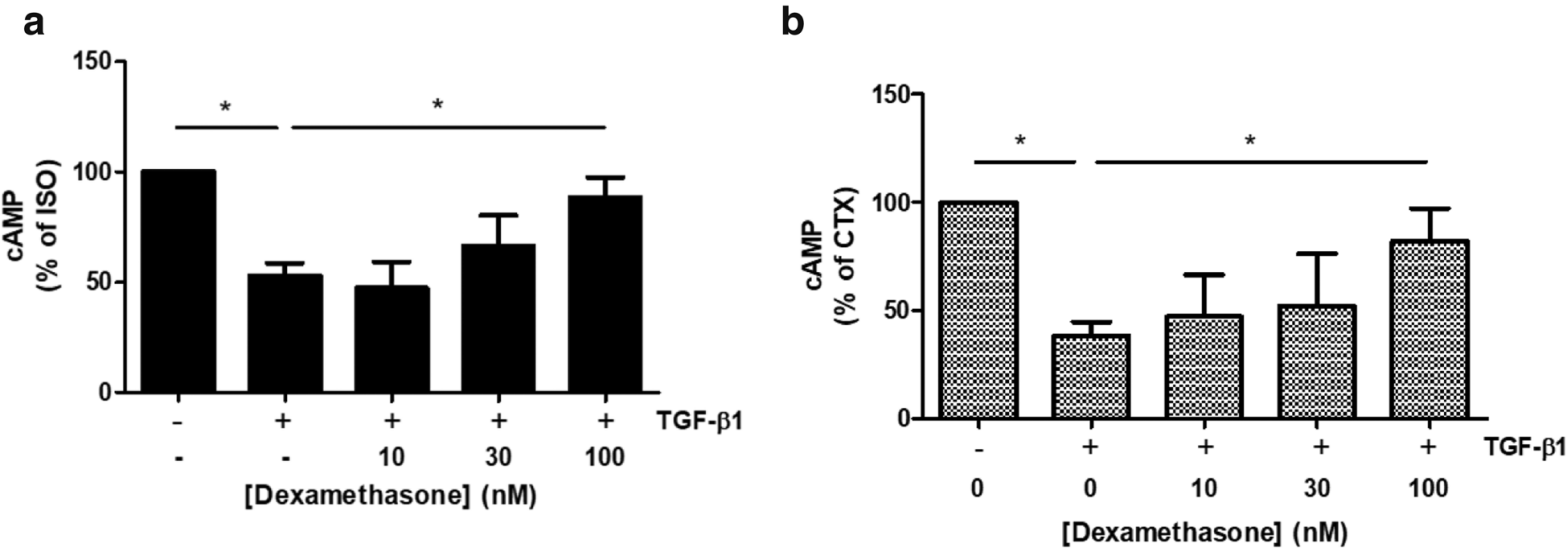
Dexamethasone (DEX) rescues TGF-β1-mediated attenuation of ISO- and CTX-induced cAMP production in HASM. HASM were pretreated with (**a**, **b**) DEX (10–100 nM, 30 min) prior to TGF-β1 (10 ng/ml, 18 h) stimulation. HASM were subsequently stimulated with (**a**) ISO (1 μM, 5 min) or (**b**) CTX (0.25 μg/ml, 45 min) and assessed for cAMP generation. Data is expressed % of max cAMP produced either by ISO (**a**) or CTX (**b**). Data is representative of (**a**) *n* = 6–13 (**b**) or *n* = 4–8 separate cell lines as mean ± SEM, *p < 0.05 comparing control/ISO/CTX to TGF-β1/ISO/CTX, and TGF-β1/ISO/CTX to TGF-β1/ISO/CTX ± DEX using a one-way ANOVA, and comparisons between two conditions assessed by Student’s t-test

Glucocorticoids remain a cornerstone in the management of asthma by decreasing airway inflammation and reversing β2AR desensitization [27, 28]. To address whether the steroids also modulate TGF-β1 effects on cAMP levels, HASM cells were pretreated with dexamethasone (DEX) in the presence and absence of TGF-β1 and then treated with either ISO or CTX. As shown in Fig. 4a, TGF-β1 treatment attenuated ISO-induced cAMP by 47.3% and DEX pretreatment significantly rescued TGF-β1’s effect by 35.7%. Similarly, TGF-β1 treatment attenuated CTX-induced cAMP by 61.7%, and DEX pretreatment significantly reversed TGF-β1’s effect by 43.8% (Fig. 4b). These data demonstrate partial steroid responsiveness of TGF-β1-mediated hyporesponsiveness to β2AR-induced cAMP levels in HASM.

### Dexamethasone attenuates TGF-β1-induced *pde4d* expression in HASM

**Fig. 5:**
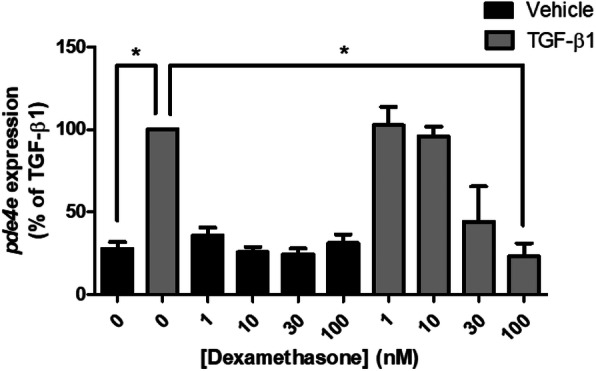
DEX inhibits TGF-β1-induced *pde4d* expression in HASM. HASM were pretreated with DEX (1–100 nM, 30 min) prior to stimulation with TGF-β1 (10 ng/ml, 18 h). mRNA was extracted, reverse transcribed, and assessed for *pde4d* and *β-actin* expression by TaqMan qPCR analysis. Data is represented as % of *pde4d* expression induced by TGF-β1, with *pde4d* expression normalized to *β-actin*. Data is representative of *n* = 4–6 separate cell lines as mean ± SEM, *p < 0.05 using a one-way ANOVA, and comparisons between two conditions assessed by Student’s t-test. TGF-β1 vs TGF-β1+ DEX (30 nM), *p* = 0.08

Previously, we determined that the blunted cAMP response to ISO induced by TGF-β1 was dependent upon increased *pde4d* expression [11]. Given that DEX rescued TGF-β1-induced attenuation of ISO- and CTX-induced cAMP production, we posited that DEX pretreatment would attenuate TGF-β1-mediated *pde4d* expression. In a dose-dependent manner, DEX pretreatment significantly attenuated TGF-β1-induced *pde4d* expression in HASM as shown in Fig. 5.

## Discussion

Insensitivity to current therapeutics occurs in some patients with severe asthma. Evidence suggests that β_2_AR dysfunction can manifest in an inflammatory milieu due to inflammatory cytokines associated with severe asthma and airway remodeling such as TGF-β1, IL-13, and TNF-α [11, 19, 29]. Additionally, TGF-β1 stimulation has been shown to attenuate β adrenergic receptor signaling in other cell types [30, 31] in addition to HASM. We examined whether generation of cAMP levels in HASM cells after CTX-treatment occurred in the presence and absence of TGF-β1 (Fig. 1b). As we had previously described, β_2_-agonist-induced responses were blunted following TGF-β1 treatment. To characterize mechanisms by which TGF-β1 diminishes β2AR responses, the effects of TGF-β1 on G_αs_-induced reversal of agonist-induced HASM cell contractile signaling was examined. We extended our previous findings that phosphorylation of MLC, an important element of agonist-induced HASM contraction, induced by Cch or TGF-β1 was reversed by activation of the β2AR and G_αs_ (Fig. 2). Given these data and our previously published results, these data suggest that the effects of TGF-β1 are more prominent at the receptor and G_αs_ level rather than downstream at the level of adenylyl cyclase, as evidenced by the lack of effect of TGF-β1 on forskolin-induced increases in cAMP levels.

Attenuation of β_2_AR signaling occurs after phosphorylation of the receptor, promoting internalization and desensitization of the β_2_AR [32–34]. Activation of GRK2 and GRK3, members of the GRK (G protein-coupled receptor kinase) family, provide a mechanism by which β_2_AR desensitization occurs. We previously demonstrated that TGF-β1 stimulation induces β_2_AR phosphorylation that is consistent with desensitization of the receptor [26]. To assess a potential role for GRKs in attenuating β_2_-agonist-induced relaxation of HASM, *grk2* and *grk3* expression were assessed with overnight TGF-β1 treatment. In Fig. 3, we demonstrate that TGF-β1 treatment decreased *grk2* and *grk3* expression. Given these data, it is highly unlikely that upregulation of GRK2/3 expression contributes the hyporesponsiveness to bronchodilators induced by TGF-β1. Current evidence and our previous studies suggest that TGF-β1 likely modulates activity of these types of kinases, rather than modulating GRK expression to induce hyporesponsiveness to β_2_-agonists.

**Fig. 6:**
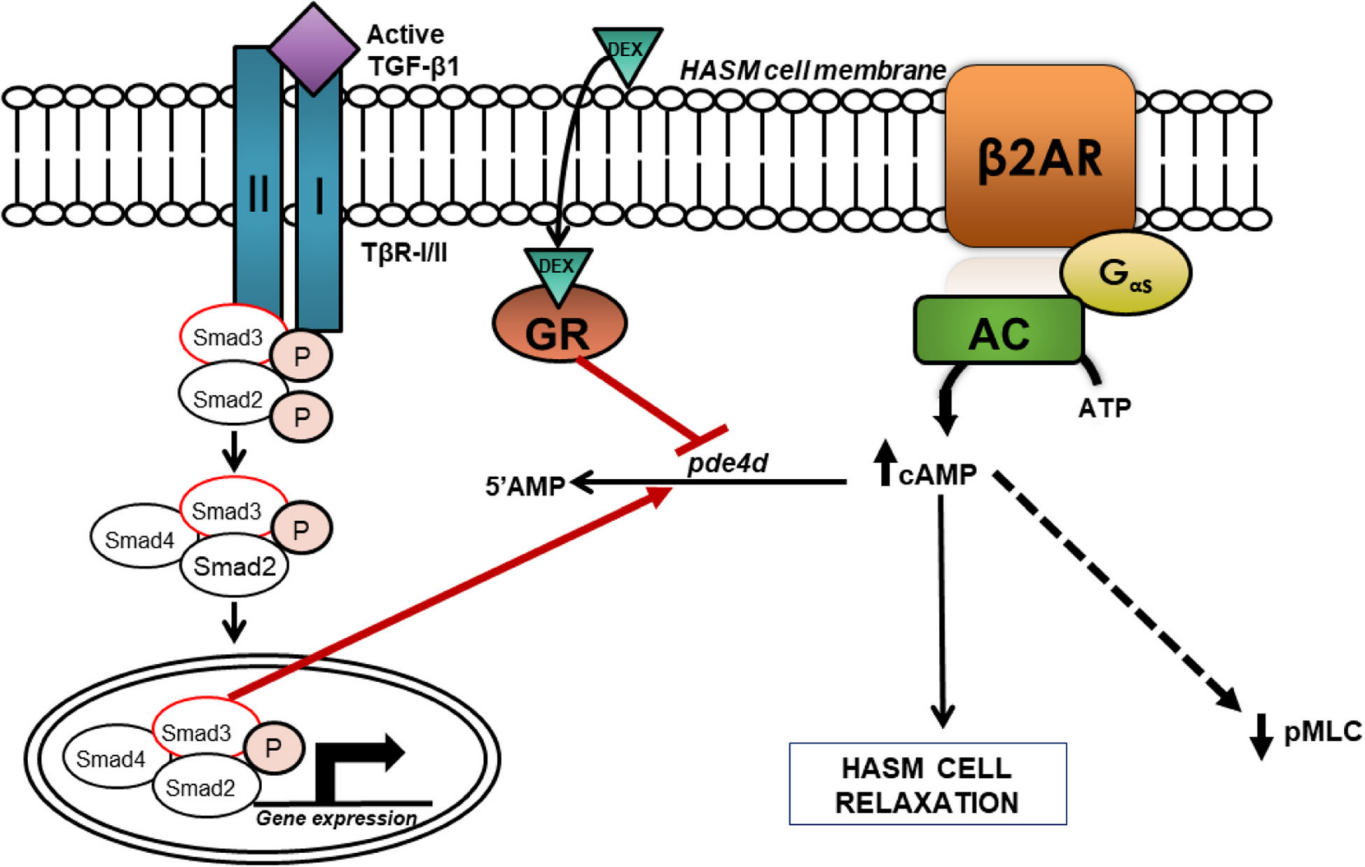
A proposed model of GC-mediated rescue of TGF-β1-induced hyporesponsiveness to bronchodilators. We previously demonstrated that TGF-β1 induces Smad2/3 activation to increase *pde4d* expression, leading to increased cAMP hydrolysis and attenuating HASM cell relaxation responses. We posited that DEX binds to the glucocorticoid receptor (GR), inhibiting both increased *pde4d* expression and rescuing TGF-β1-induced attenuation of β_2_AR and G_αs_-induced cAMP production. AC = Adenylyl Cyclase; β_2_AR = β_2_-adrenergic receptor; DEX = Dexamethasone; G_αs_ = Stimulatory G_α_ protein; GR = Glucocorticoid Receptor; PDE4D = Phosphodiesterase 4D; TBR-I/II = TGF-β receptor I/II; pMLC = phosphorylated myosin light chain

Currently, glucocorticoids like dexamethasone are used to attenuate inflammation associated with asthma as well as restore β_2_AR responsiveness [3, 19, 35], and reverse the effects of inflammatory mediator-induced β_2_AR dysfunction. In osteoblasts, it has been demonstrated that dexamethasone suppresses the transcriptional activity of, but not expression, Smad3, attenuating TGF-β1-induced alkaline phosphatase activity and type I collagen expression [16]. Additionally, dexamethasone also repressed transcriptional activation of PAI-1 through inhibition of Smad3/4 by direct interaction between the glucocorticoid receptor and Smad3 [17]. We previously showed that TGF-β1-induced attenuation of ISO reversal of Cch-induced pMLC was Smad3-dependent in HASM. We also showed that TGF-β1 treatment increased expression of *pde4d*, suggesting a role for phosphodiesterases in TGF-β1-induced hyporesponsiveness to bronchodilators. Given our data and others’ observations concerning the effects of glucocorticoids on TGF-β1/Smad3-dependent signaling, we posited that dexamethasone would thereby reverse TGF-β1- and Smad3-induced attenuation of ISO-induced cAMP accumulation and *pde4d* expression in HASM. Figure 6 depicts a model of mechanisms underlying glucocorticoid-mediated rescue of TGF-β1-induced β_2_AR hyporesponsiveness. We demonstrate that TGF-β1-induced attenuation of β_2_-agonist- and G_αs_-induced cAMP accumulation in HASM can be rescued by treatment with dexamethasone (Fig. 4a & b). Our data also show that DEX treatment reversed TGF-β1-induced *pde4d* expression (Fig. 5). Consistent with these data, we previously demonstrated that DEX (1 uM, 18 h) stimulation alone has little effect *pde4d* expression [36]. We recognize that changes in transcript expression do not necessarily translate to changes in protein expression or PDE4D activity. Previous work in HASM has demonstrated that PDE4D5 is expressed and, in part, controls cAMP production following β_2_ agonist stimulation [37, 38]. As in our study, Billington et al. demonstrated transcript expression, but not protein expression or enzymatic activity, of the specific PDE isoform. Interestingly, Niimi et al. demonstrated that transcript expression of PDE4D isoforms in HASM was altered, but not protein or enzymatic activity [39]. Extensive work by the Houslay group has shown the difficulty of isolating specific isoforms and splice variants of PDE enzymes, as well as the difficulty of enzymatic activity assessment [40]. Given the findings of Billington, Niimi, and Trian, and the complexities of the assays performed by the Houslay group, we recognize the limitations to our work, but feel that our results likely translate to functional outcomes affecting bronchomotor tone in the context of TGF-β1-induced hyposensitivity to β_2_ agonists and cAMP mobilizers.

Despite the fact that we showed that TGF-β1 attenuates CTX-induced cAMP production, and that DEX rescues TGF-β1-mediated attenuation of both ISO- and CTX-induced cAMP production, it remains to be seen if these mechanisms are operative in vivo. Despite this limitation, we have shown effects of TGF-β1 on HASM to be recapitulated in small airways derived from human lungs [10]. Additionally, while it would be interesting to study this phenomenon in asthma-derived HASM, we and others have demonstrated that β_2_-agonist-induced cAMP production in these cells is already blunted due partially to increased PDE expression [26, 38]. Therefore, exposure of asthma-derived HASM to TGF-β1 will likely have little effect on modulating β_2_-agonist-induced cAMP production.

## Conclusion

Regardless of evidence that steroids may not reverse the TGF-β1-induced remodeling effects [41] in asthma, our findings suggest that in asthma patients with high levels of TGF-β1, steroids may be an effective treatment to reverse β_2_AR hyporesponsiveness observed in these patients.

## Supplementary information

**Additional file 1: Table S1.** Donor demographics for cAMP and *pde4d* expression studies. All cells were derived from subjects with no history of chronic disease.

## Data Availability

All data and materials that support the findings of this study are available from the corresponding author upon reasonable request.

## Abbreviations

TGF-β1: Transforming growth factor β1
GC: Glucocorticoid
β_2_AR: Beta 2 adrenergic receptor
HASM: Human airway smooth muscle
PDE4D: Phosphodiesterase 4D
DEX: Dexamethasone
ISO: Isoproterenol
CTX: Cholera toxin
GRK: G protein receptor-coupled kinase
ASM: Airway smooth muscle
Smad3: Mothers against decapentaplegic homolog 3
cAMP: Adenosine 3′,5′-cyclic monophosphate
GPCR: G protein-coupled receptor
ADP: Adenosine monophosphate
PKA: Protein kinase A
AC: Adenylyl cyclase
PBS: Phosphate buffered saline
pMLC: Phosphorylated myosin light chain
MLC: Myosin light chain
GAPDH: Glyceraldehyde-3-phosphate dehydrogenase
cAMP-AP: Adenosine 3′,5′-cyclic monophosphate-alkaline phosphatase
CSPD: Chemiluminescent alkaline phosphatase substrate
RNA: Ribonucleic acid
qPCR: Quantitative polymerase chain reaction
mRNA: Messenger ribonucleic acid
cDNA: Complementary deoxyribonucleic acid
ANOVA: Analysis of variance
Alb: Albuterol
Cch: Carbachol
FSK: Forskolin
IL-13: Interleukin 13
TNFα: Tumor necrosis factor α
