# Supplementary material for: Dexamethasone rescues TGF-β1-mediated β2-adrenergic receptor dysfunction and attenuates phosphodiesterase 4D expression in human airway smooth muscle cells
Source: Respir Res. 2020 Oct 8;21:256. doi: 10.1186/s12931-020-01522-w (PMC7545943; doi:10.1186/s12931-020-01522-w)

Donor Characteristics*	
Sex, M/F	27/14
Age, y/o	30.88 (14.07)
Race, C/B/H	28/7/6
BMI, kg/m^3	26.88 (6.74)

Data represents means (SD); n=41 donors. M, male; F, female;
C, caucasian; B, black; H, Hispanic; BMI, body mass index
* Donors died of head trauma or cardiovascular incident, including stroke

Table S1: Donor demographics for cAMP and *pde4d* expression studies. All cells were derived from subjects with no history of chronic disease.

Figure 1

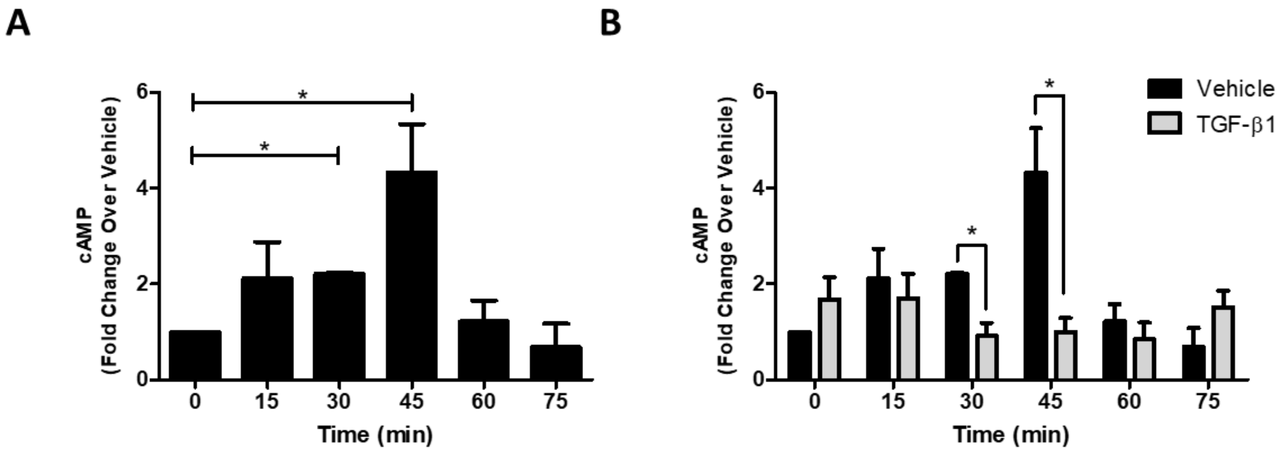

Figure 2

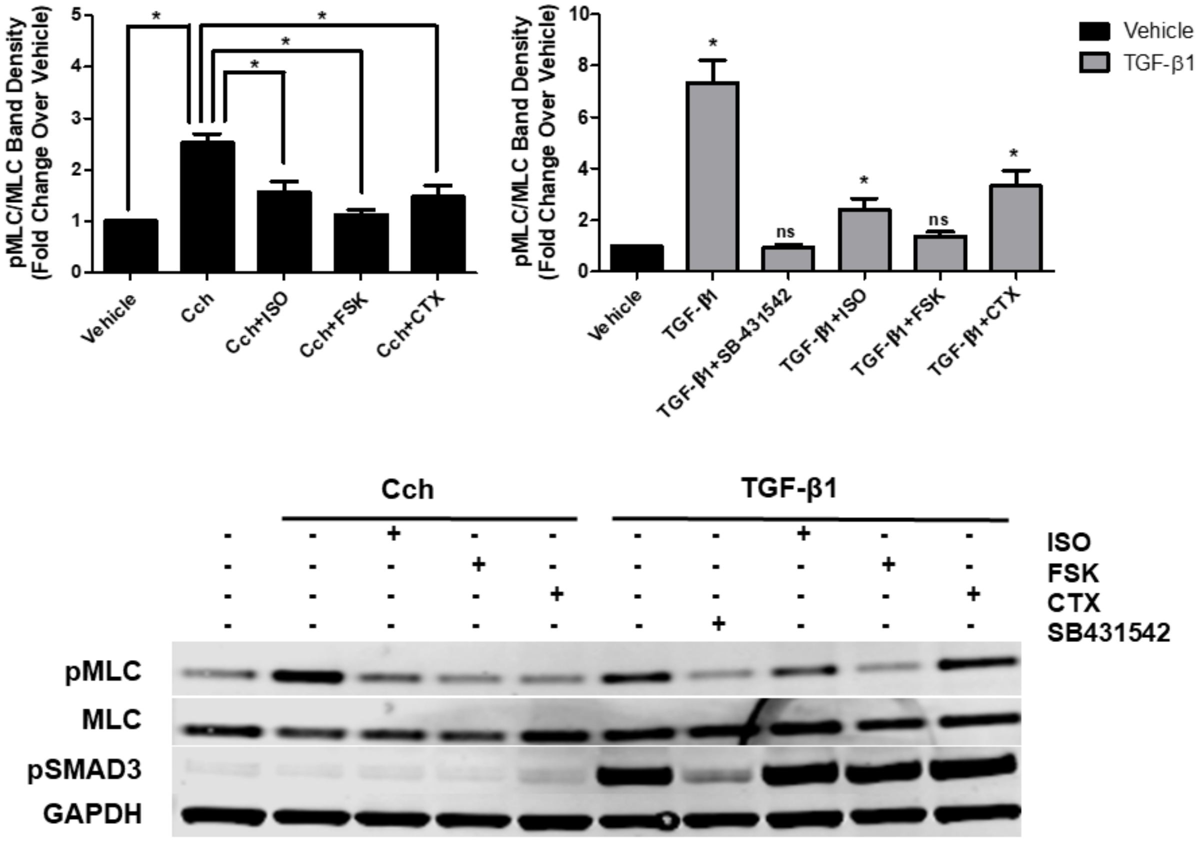

Figure 3

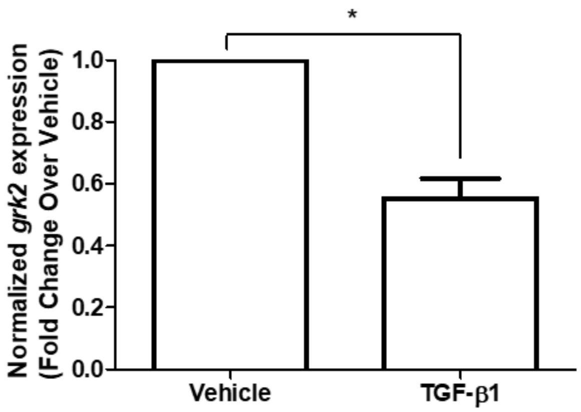

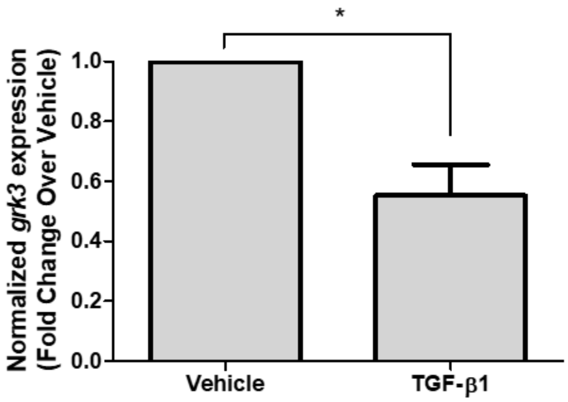

Figure 4

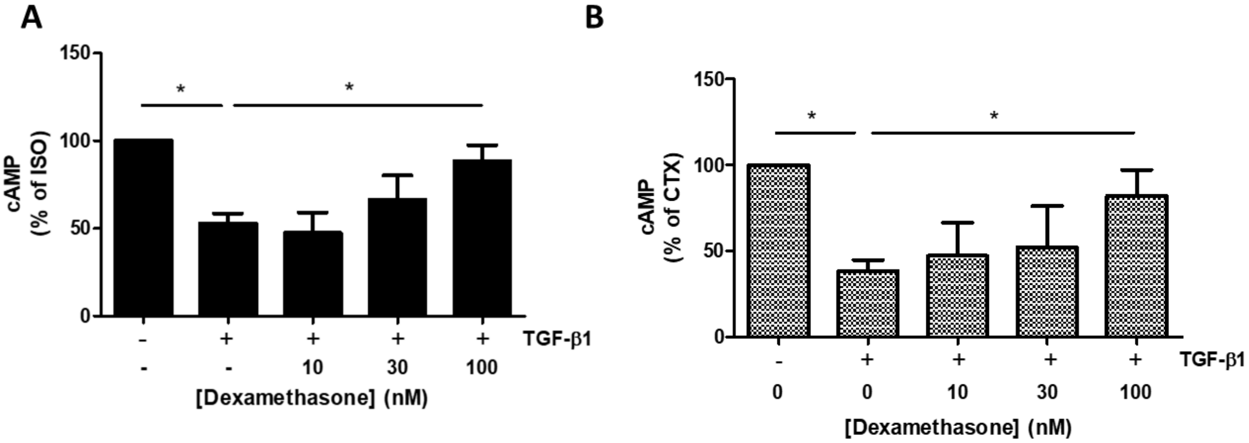

Figure 5

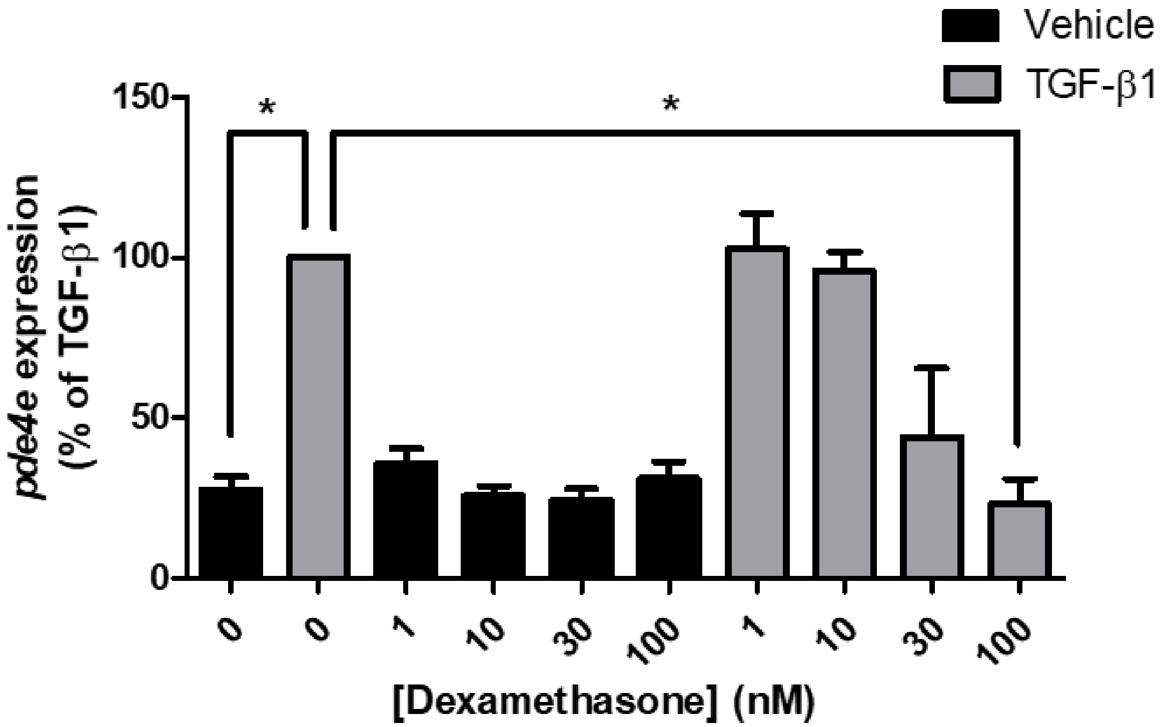

Figure 6

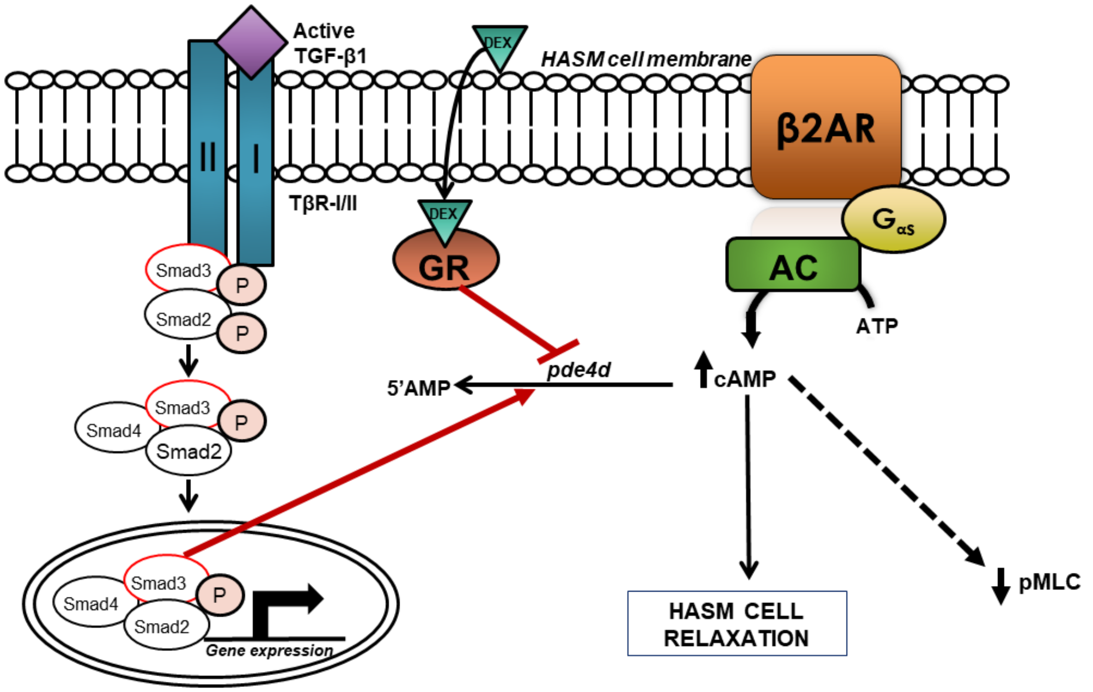

Supplement: Supplementary file 1 — Additional file 1: Table S1. Donor demographics for cAMP and pde4d expression studies. All cells were derived from subjects with no history of chronic disease. [file 12931_2020_1522_MOESM1_ESM.pdf]
